# Supplementary material for: IL-23 signaling is not an important driver of liver inflammation and fibrosis in murine non-alcoholic steatohepatitis models
Source: PLoS One. 2022 Sep 15;17(9):e0274582. doi: 10.1371/journal.pone.0274582 (PMC9477333; doi:10.1371/journal.pone.0274582)
Supplement: Attachment — Submitted filename: PLOS_ONE_IL23_PT_Comments.pdf [file pone.0274582.s002.pdf]

PLOS ONE

IL23 signaling is not an important driver of liver inflammation and fibrosis in murine non-alcoholic steatohepatitis models

--Manuscript Draft--

**Manuscript Number:**	PONE-D-22-09562
**Article Type:**	Research Article
**Full Title:**	IL23 signaling is not an important driver of liver inflammation and fibrosis in murine non-alcoholic steatohepatitis models
**Short Title:**	IL23 is not required for NASH
**Corresponding Author:**	Ning Ding<br>Genentech USA Inc South San Francisco<br>South San Francisco, California UNITED STATES
**Keywords:**	
**Abstract:**	Non-alcoholic fatty liver disease (NAFLD), represents an unmet medical need that can progress to non-alcoholic steatohepatitis (NASH), which, without intervention, can result in the development of cirrhosis and hepatocellular carcinoma (HCC). Inflammation is a pathological hallmark of NASH, and targeting key inflammatory mediators of NASH may lead to potential therapeutics for the disease. Herein, we aimed to investigate the role of IL-23 signaling in the disease progression in murine NASH models. We showed that recombinant IL-23 can promote IL-17 producing cell expansion in the liver and that these cells are predominately T cells and Mucosal Associated Invariant T cells (MAITs). Reciprocally, we found that IL23R is necessary for the expansion of T cells and MAIT cells in the western diet (WD) diet induced NASH model. However, we did not observe any dramatic differences in liver inflammation and fibrosis between wild type and Il23r-/- mice in the same NASH model. Furthermore, we found that Il23r deletion does not impact liver inflammation and fibrosis in the choline-deficient, L-amino acid-defined and high-fat diet (CDA-HFD) induced NASH model. Based on these findings, we therefore propose that IL-23 signaling is not a crucial determinant of NASH pathogenesis in preclinical models and targeting this pathway may not be sufficient to ameliorate the disease progression in NASH patients.
**Order of Authors:**	Ning Ding
	Jose E. Heredia
	Clare Sorenson
	Sean Flanagan
	Victor Nunez
	Charles Jones
	Angela Martzall
	Laurie Leong
	Andres Martinez-Paler
	Alexis Scherl
	Hans D. Brightbill
	Nico Ghilardi
**Additional Information:**	
**Question**	**Response**
**Financial Disclosure**<br><br>Enter a financial disclosure statement that	The author(s) received no specific funding for this work.

describes the sources of funding for the work included in this submission. Review the submission guidelines for detailed requirements. View published research articles from *PLOS ONE* for specific examples.<br><br>This statement is required for submission and **will appear in the published article** if the submission is accepted. Please make sure it is accurate.<br><br>**Unfunded studies**<br>Enter: *The author(s) received no specific funding for this work.*<br><br>**Funded studies**<br>Enter a statement with the following details:<br>• Initials of the authors who received each award<br>• Grant numbers awarded to each author<br>• The full name of each funder<br>• URL of each funder website<br>• Did the sponsors or funders play any role in the study design, data collection and analysis, decision to publish, or preparation of the manuscript?<br>• **NO** - Include this sentence at the end of your statement: *The funders had no role in study design, data collection and analysis, decision to publish, or preparation of the manuscript.*<br>• **YES** - Specify the role(s) played.<br><br>* typeset	
**Competing Interests**<br><br>Use the instructions below to enter a competing interest statement for this submission. On behalf of all authors, disclose any competing interests that could be perceived to bias this work—acknowledging all financial support and any other relevant financial or non-financial competing interests.<br><br>This statement is required for submission and **will appear in the published article** if the submission is accepted. Please make sure it is accurate and that any funding	All authors are or were employees of Genentech/Roche

sources listed in your Funding Information later in the submission form are also declared in your Financial Disclosure statement.<br><br>View published research articles from *PLOS ONE* for specific examples.<br><br>**NO authors have competing interests**<br><br>Enter: *The authors have declared that no competing interests exist.*<br><br>**Authors with competing interests**<br><br>Enter competing interest details beginning with this statement:<br><br>*I have read the journal's policy and the authors of this manuscript have the following competing interests: [insert competing interests here]*<br><br>* typeset	
**Ethics Statement**<br><br>Enter an ethics statement for this submission. This statement is required if the study involved:<br><br>• Human participants<br>• Human specimens or tissue<br>• Vertebrate animals or cephalopods<br>• Vertebrate embryos or tissues<br>• Field research<br><br>Write "N/A" if the submission does not require an ethics statement.<br><br>General guidance is provided below. Consult the submission guidelines for detailed instructions. **Make sure that all information entered here is included in the Methods section of the manuscript.**	NA

**Format for specific study types**<br><br>**Human Subject Research (involving human participants and/or tissue)**<br>• Give the name of the institutional review board or ethics committee that approved the study<br>• Include the approval number and/or a statement indicating approval of this research<br>• Indicate the form of consent obtained (written/oral) or the reason that consent was not obtained (e.g. the data were analyzed anonymously)<br><br>**Animal Research (involving vertebrate animals, embryos or tissues)**<br>• Provide the name of the Institutional Animal Care and Use Committee (IACUC) or other relevant ethics board that reviewed the study protocol, and indicate whether they approved this research or granted a formal waiver of ethical approval<br>• Include an approval number if one was obtained<br>• If the study involved *non-human primates*, add *additional details* about animal welfare and steps taken to ameliorate suffering<br>• If anesthesia, euthanasia, or any kind of animal sacrifice is part of the study, include briefly which substances and/or methods were applied<br><br>**Field Research**<br><br>Include the following details if this study involves the collection of plant, animal, or other materials from a natural setting:<br>• Field permit number<br>• Name of the institution or relevant body that granted permission	
**Data Availability**<br><br>Authors are required to make all data underlying the findings described fully available, without restriction, and from the time of publication. PLOS allows rare exceptions to address legal and ethical concerns. See the PLOS Data Policy and FAQ for detailed information.	Yes - all data are fully available without restriction

Powered by Editorial Manager® and ProduXion Manager® from Aries Systems Corporation

A Data Availability Statement describing where the data can be found is required at submission. Your answers to this question constitute the Data Availability Statement and **will be published in the article**, if accepted.<br><br>**Important:** Stating 'data available on request from the author' is not sufficient. If your data are only available upon request, select 'No' for the first question and explain your exceptional situation in the text box.<br><br>Do the authors confirm that all data underlying the findings described in their manuscript are fully available without restriction?	
**Describe where the data may be found in full sentences. If you are copying our sample text, replace any instances of XXX with the appropriate details.**<br><br>• If the data are **held or will be held in a public repository**, include URLs, accession numbers or DOIs. If this information will only be available after acceptance, indicate this by ticking the box below. For example: *All XXX files are available from the XXX database (accession number(s) XXX, XXX.).*<br>• If the data are all contained **within the manuscript and/or Supporting Information files**, enter the following: *All relevant data are within the manuscript and its Supporting Information files.*<br>• If neither of these applies but you are able to provide **details of access elsewhere**, with or without limitations, please do so. For example:<br><br>*Data cannot be shared publicly because of [XXX]. Data are available from the XXX Institutional Data Access / Ethics Committee (contact via XXX) for researchers who meet the criteria for access to confidential data.*<br><br>*The data underlying the results presented in the study are available from (include the name of the third party*	All relevant data are within the manuscript and its Supporting Information files.

and contact information or URL).<br>• This text is appropriate if the data are owned by a third party and authors do not have permission to share the data.<br><br>* typeset	
Additional data availability information:	

Powered by Editorial Manager® and ProduXion Manager® from Aries Systems Corporation

manuscript and figures

Click here to access/download;Manuscript;il23 NASH.pdf

IL23 signaling is not an important driver of liver inflammation and fibrosis in murine non-alcoholic steatohepatitis models

Authors

Jose E. Heredia[1]†, Clare Sorenson[2], Sean Flanagan[2], Victor Nunez[2], Charles Jones[2], Angela Martzall[2], Laurie Leong[2], Andres Martinez-Paler[1], Alexis Scherl[2], Hans D. Brightbill[3], Nico Ghilardi[1*], Ning Ding[1]†

Affiliation

[1] Department of Discovery Immunology, Genentech, South San Francisco, CA, USA

[2] Department of Pathology, Genentech, South San Francisco, CA, USA

[3] Department of Translational Immunology, Genentech, South San Francisco, CA, USA

* Current address: Dice Therapeutics, South San Francisco, CA, USA.

† To whom correspondence should be addressed: heredij1@gene.com or ding.ning@gene.com

Abstract

Non-alcoholic fatty liver disease (NAFLD), represents an unmet medical need that can progress to non-alcoholic steatohepatitis (NASH), which, without intervention, can result in the development of cirrhosis and hepatocellular carcinoma (HCC). Inflammation is a pathological hallmark of NASH, and targeting key inflammatory mediators of NASH may lead to potential therapeutics for the disease. Herein, we aimed to investigate the role of IL-23 signaling in the disease progression in murine NASH models. We showed that recombinant IL-23 can promote IL-

17 producing cell expansion in the liver and that these cells are predominately $\gamma\delta$ T cells and Mucosal Associated Invariant T cells (MAITs). Reciprocally, we found that IL23R is necessary for the expansion of $\gamma\delta$ T cells and MAIT cells in the western diet (WD) diet induced NASH model. However, we did not observe any dramatic differences in liver inflammation and fibrosis between wild type and Il23r-/- mice in the same NASH model. Furthermore, we found that Il23r deletion does not impact liver inflammation and fibrosis in the choline-deficient, L-amino acid-defined and high-fat diet (CDA-HFD) induced NASH model. Based on these findings, we therefore propose that IL-23 signaling is not a crucial determinant of NASH pathogenesis in preclinical models and targeting this pathway may not be sufficient to ameliorate the disease progression in NASH patients.

Introduction

Non-alcoholic fatty liver disease (NAFLD) is defined as a chronic liver disease that imparts excess lipid accumulation in the liver in the absence of secondary causes such as viral infections or alcohol abuse[1, 2]. NALFD can progress from simple hepatic steatosis to non-alcoholic steatohepatitis (NASH) characterized by inflammation and fibrosis, which is a significant risk factor for cirrhosis and hepatocellular carcinoma (HCC) [3-6]. Within the past decades, the epidemic of obesity has led to the sharp rise of NALFD/NASH occurrence [2, 7]. However, there are no FDA-approved therapies for NASH driven chronic liver disease, which may be largely due to our limited understanding of molecular underpinnings of liver inflammation and fibrosis.

IL-23 is a cytokine that has been implicated in IL-17 driven pathologies such psoriasis, colitis, and autoimmune diseases [8-11]. As an IL-12 cytokine family member, IL-23 is composed of a heterodimer of IL-12p40 subunit and IL-23p19 subunit (IL-23p19/p40) which signals through the IL-23R and IL-12Rβ1 dimeric receptor [12]. Mice that lack IL-23p19 demonstrate an inability to drive the expansion of pathogenic IL-17 producing cells [13-15]. All IL-17 expressing cells also express RAR-related orphan receptor gamma t (RORγt), the master transcription factor driving the TH17 signature [16, 17]. In this regard, previous studies have implicated that IL-17 producing cells promote hepatic inflammation and fibrosis[18]. It has also been reported that there is accumulation of IL-17 producing cells in the livers from NASH patients or diet induced NASH preclinical murine models [19-22]. Additionally, IL-17 has been shown to be elevated by liver damaging agents such as Carbon Tetra-Chloride and Concanavalin A in models of acute hepatitis [18, 20-23]. While these studies suggest that IL-17 producing cells may contribute to chronic liver disease, the role of IL-23 signaling in NASH has not been fully dissected.

In this study, we hypothesized that IL-23 signaling may play an important role in the NASH pathogenesis. We showed that systemic administration of recombinant IL-23 protein induces IL-17 producing cell expansion in the liver and that these cells are predominately $\gamma\delta$ T cells and Mucosal Associated Invariant T cells (MAITs). Reciprocally, genetic ablation of Il23r attenuated $\gamma\delta$ T and MAIT cell expansion in western diet (WD)-induced NASH model. However, we did not observe that Il23r deletion exhibits dramatic effects on liver inflammation, fibrosis and liver function in the same model. Similarly, we found that Il23r-/- mice are not protected from liver inflammation and fibrosis in another model, the choline-deficient, L-amino acid-defined and

high-fat diet (CDA-HFD) induced NASH model. Thus, these results do not support a causal role of IL-23 signaling in the NASH pathogenesis and suggest that targeting IL-23 signaling may not be a viable therapeutic strategy to treat NASH patients.

Results

Recombinant IL-23 increases RORγt cell accumulation in liver

In order to determine whether IL-23 is sufficient to induce hepatic expansion of RORγt positive IL-17 producing cells, we intraperitoneally injected 0.5ug recombinant mouse IL-23 (rmIL-23) daily for three consecutive days into mice fed on normal diet (ND) and analyzed the livers 24hrs after the last injection. Administration of rmIL-23 led to a five-fold expansion of hepatic Ki67$^+$ RORγt cells (Figures 1A and 1B) and a two-fold increase in the percentage of Ki67$^+$ RORγt cells when compared to vehicle control (Figure 1C). RORγt cells that proliferated actively were identified as MAIT and γδ T Cells (Figures 1D and 1E). rmIL-23 treatment also induced an increase in the percentage of hepatic neutrophils and inflammatory monocytes when compared to vehicle control (Figures 1F and 1G). These results thus suggest that IL-23 is sufficient to induce RORγt cell accumulation and pro-inflammatory response within the livers.

Western Diet induced hepatic expansion of RORγt cells is dependent on IL-23R

Next, we investigated whether IL-23 signaling is required for of RORγt cell accumulation in the animal model of NASH. Western Diet (WD) consisting of high fat, high fructose, and added cholesterol have been established to induce several NASH phenotypes including hepatic inflammation, fibrosis and an increase in liver damage measured by the serum biomarkers such

as alanine aminotransferase (ALT), aspartate aminotransferase (AST), all in a nutritional setting without liver damaging chemicals [24, 25]. We fed WT and Il23r-/- mice with normal diet (ND) or WD for 20 weeks (Figure 2A) [26]. The WD induced the expansion of RORγt^{+} $\gamma\delta$ T cells and MAIT cells in livers from WT mice (Figure 2B and 2C). In Il23r-/- mice, we found that WD-induced expansion of $\gamma\delta$ T cells and MAIT cells were dramatically normalized to the baseline. Furthermore, by intracellular staining of liver $\gamma\delta$ T cells, we observed a significant decrease of IL-17A production from Il23r-/- compared to WT. These data suggest that IL-23 plays an important role in regulating IL17 producing cells in WD induced NASH model.

IL-23 signaling is not critical for hepatic inflammation and fibrosis induced by WD in mice

Having established its critical role in WD-indued RORγt cell accumulation, we explored the contribution of IL-23 signaling to WD-induced liver inflammation and fibrosis. While there is a clear increase of hepatic inflammation induced by WD, we did not observe any noticeable differences in liver inflammation between WT and Il23r-/- livers from WD fed mice as assessed by histology and pro-inflammatory gene expression (Figures 3A and 3B). On the other hand, while we observed a modest, but statistically significant, reduction of pro-inflammatory monocytes in the WD indued Il23r-/- liver (Figure 3C), there was no significant difference of neutrophil infiltration to the liver between Il23-/- and WT mice (Figure 3D), the main myeloid cell known to be recruited by IL-17 induced chemokines. In addition, we found that the serum levels of Keratinocytes-derived chemokine (KC) (Figure 3E) and Interferon gamma-induced protein 10 (IP-10) (Figure 3F) were not changed in Il23r-/- mice. Next, to address the role of IL23R signaling in liver fibrosis in the WD-induced NASH model, we evaluated hepatic collagen content by

trichrome staining analysis and hepatic collagen gene expression. We did not observe a significant difference of collagen content at the histology level as well as at the transcriptional level (Figures 4A and 4B). Overall, these results do not suggest IL-23 signaling as the main driver of liver inflammation and fibrosis in the WD-induced NASH model.

IL-23 signaling does not contribute to WD induced liver dysfunction

Next, we examined the impact of Il23r deletion on liver function. In this regard, we measured several serum biomarkers of liver function. The results showed that there is little difference of serum ALT/AST, alkaline phosphatase (AP), albumin, cholesterol, and triglyceride levels between WT and Il23r-/- mice fed on WD (Figure 5C-5H). Similarly, IL-23R deficiency appears not to affect the WD-induced whole-body weight as well as liver weight gains (Figure 5A and 5B). Collectively, these data suggests that IL-23 signaling may not contribute to liver dysfunction caused by WD-induced metabolic imbalance.

IL-23 signaling does not contribute to liver inflammation and fibrosis in the CDA-HFD model of NASH

To complement our findings in WD-induced NASH model, we sought to determine whether IL23 signaling contributes to the pathogenies of NASH in another animal model. In this regard, we chose the CDA-HFD model (Figure 6A) because this model has been demonstrated to recapitulate steatosis, inflammation, and advanced fibrosis in liver [27]. The CDA-HFD significantly induced the expansion of RORγ+ $\gamma\delta$ T Cells and MAIT cells in mouse livers (Figures 6B and 6C). Consistent to our observation in WD-induced NASH model, we found no impact of IL-23R depletion on liver

inflammation and fibrosis by histology (Figures 6D). Furthermore, no significant changes in hepatic inflammatory or fibrotic gene expression were detected between WT and Il23r-/- mice fed on CDA-HFD (Figure 6G). In addition, we did not observe any significant change in body weight, liver weight, and a variety of serum biomarkers for liver function between WT and Il23r-/- mice. Taken together, these results suggest that IL-23 signaling does not contribute to hepatic inflammation and fibrosis in the CDA-HFD NASH model.

Discussion

NAFLD/NASH is an unmet medical need that is increasingly common around the world. The incidence of NAFLD world-wide is approximately 25%, and the global prevalence of NASH patients from NAFLD biopsied patients was estimated to be 59.1% based on Meta-analysis data [7, 28]. There are currently no approved therapies for NAFLD/NASH, and pro-inflammatory pathways have been proposed to be a class of appealing targets for this complex disease [29]. In this regard, it came to our attention that hepatic IL-17 producing cells have been shown to promote liver inflammation and dysfunction [18-21]. However, genetic dissection of this pathway, particularly its upstream regulator IL-23, in the preclinical NASH models is lacking and the target candidacy of this Il-17/Il-23 axis in NASH is yet to be fully established.

In this context, we therefore chose to investigate the contribution of Il-23 signaling to NASH pathogenesis by testing IL-23R deficient mice in animal models of NASH. Our data showed that, while recombinant IL-23 is sufficient to drive IL-17A producing cell expansion and pro-inflammatory myeloid cell infiltration in liver, Il-23r-/- mice are not protected from liver

inflammation and fibrosis in two NASH models, suggesting the contribution of Il-23 signaling to NASH pathogenesis is minimal. These observations thus challenge the assumption that IL-17 producing cells that have been shown to be present in NASH patient liver samples may play a causal role in the disease pathogenesis[30]. It should also be noted that we cannot rule out IL-23's contribution to non-NASH liver fibrosis as some reports suggest IL-23 signaling plays a role in cholestatic or viral driven liver fibrosis [18, 31]. Nevertheless, the dispensability of IL-23 signaling in NASH driven liver inflammation is intriguing given its critical role in a wide variety of pro-inflammatory diseases. Since it is well documented that many inflammatory factors such as cytokines and PAMPs are elevated in NASH models, it is not inconceivable that the accumulation of these factors may mask any effects of IL-23 in the NASH models [29, 32]. Further studies are warranted to dissect the potential crosstalk between IL-23 and other proinflammatory cytokines during the pathogenesis of NASH.

In summary, we present the evidence that IL-23r -/- mice are not protected from liv[illegible] inflammation and fibrosis in two NASH preclinical models, thus suggesting that IL-23 signaling may not be an effective therapeutic target for NASH. Our study also supports the necessity of leveraging genetic models to validate drug targets when possible and the notion that the overall role of IL-23/IL-17 axis in NASH may need to be re-evaluated.

Material and Methods

Mouse studies

Il23r-/- mice were generated as described previously [33], the control group were littermate wild type mice. Diets used in this study were purchased from Research Diets; Normal Diet (ND) was compared either to Western Diet (WD) (Diet #D19021501) composed of 40% kcal Fat, 22% kcal Fructose, and 1.25% Cholesterol or Choline Deficient L-Amino Acid Derived High Fat Diet (CDA-HFD) (Diet #A06071302) composed of 60% kcal Fat, 0.1% Methionine, and no added choline. All mice started the diets at 8 weeks of age, and all mice used were males. The ND vs WD cohort were challenged with the diet for 20 weeks. The ND vs CDA-HFD cohorts were on the diets for 9 weeks. C57Blk/6 mice from Jackson Laboratory were used for the Intraperitoneal (IP) injects of PBS vs IL-23. Recombinant murine IL-23 was purchase from R&D, mice were IP with either PBS or 0.5ug IL-23 for three consecutive days and livers were harvested 24hrs after last injection. All animal activity were performed as required by the Institutional Animal Care and Use Committee (IACUC) of Genentech Inc., Animal Welfare Act, and in Accordance with the Guide for the care and use of laboratory animals (The Guide).

Liver digestion and flow cytometry

Upon CO_2 euthanasia, serum was collected, and livers were perfused with 1X PBS, via the portal vein. Livers were collected for either histology, snap frozen, or tissue processing for non-parenchymal cell (NPC) isolation. All liver samples per cohort were processed at the same time by transferring the livers in c-tubes (Miltenyi) and adding 5mL of digestion media consisting of 0.2% Collagenase Type 2 (Worthington), 0.1% DNAse I (Roche), 1% BSA (Sigma), in RPMI. Samples were digested using MACS Miltenyi Dissociator using liver dissociation settings, and incubating samples at 37degrees C for 30mins in shaker at 120rpm. After digestion incubation, samples were

centrifuged at 1600rpm for 5mins, ACK treated pellets were resuspended in 1X PBS and passed through a 70um cell strainer. Samples were pelleted, and resuspend in 15% Percoll, centrifuged for 1600rpm for 15mins without brake. These pellets were the non-parenchymal cell (NPC) fraction, free of hepatocytes. The NPCs were then resuspended in 1X PBS, stained with LIVE/DEAD fixable dye (Invitrogen) at a 1:1000 dilution, incubated on ice for 15mins, washed, resuspended in FACS buffer (PBS + 2.5mM EDTA + 5% BSA) with FcR block (Miltenyi), and stained with the appropriate conjugated fluro-antibodies. For RORgt staining, cells were processed/stained using the FOXP3 Transcription Factor staining kit (BD). For intracellular staining of IL-17a, NPCs were stimulated with Leukocyte Activation Cocktail with GolgiPlug (BD) for 4hours in RPMI media, then cells were washed, FcR blocked, and stained with appropriate antibodies. Samples were run and analyzed on Symphony analyzer (BD).

RNA Extraction and Quantitative PCR

RNA was isolated from approximately 100mg of liver tissue using 1mL Trizol using the bead homogenizer Qiagen method, followed by addition of 200uL chloroform, resuspended samples were centrifuged for 10mins at 13krpm, 300uL clear top aqueous layer was transferred to new tube followed by the addition of 300uL 70% Ethanol. The 600uL samples were then loaded on a RNeasy Mini purification column (Qiagen) for RNA isolation. RNA quantification and purity was analyzed with NanoDrop 2000 (Thermo Scientific). 1ug of RNA was used for cDNA synthesis using Iscript First Strand cDNA kit (BioRad). cDNA templates were combined with Taqman probes (Thermo), and Taqman Universial PCR Master Mix (Thermo), and run on QuantaStudio 6 Flex (Applied Biosystems).

Histology

Paraffin embedded liver tissues were sectioned and stained for Hematoxylin and Eosin staining and for Trichrome staining. Automated image analysis was conducted on trichrome stained slides to access fibrosis and inflammation. Features counted towards inflammation include inflammatory cells (lobular inflammation), primarily macrophages with some neutrophils, and areas of hepatocyte injury/ductular reaction. The trichrome stain allowed for robust identification of both fibrosis and inflammatory features.

Serum Biomarker and Cytokine Analysis

The liver chemistry panel consists of the following assays: Alanine Transaminase (ALT), Aspartate Transaminase (AST), Alkaline Phosphatase (AP), Albumin (ALB), and Triglycerides (TRIG). All assays were performed on the Beckman Coulter Au480 chemistry analyzer using the analytical principle of spectrophotometry and potentiometry. (Beckman Coulter Inc., Brea CA). Serum cytokines were measured using Luminex bead assay (Millipore platform).

Quantification and Statistical Analysis

GraphPad Prism 6 was used for statistical methods using the unpaired student t-test for panels in Figure 1 and Figure 2D. All other experiments were performed using one-way ANOVA. Statistical details provided in the figure legends.

References

1. Powell EE, Wong VW, Rinella M. Non-alcoholic fatty liver disease. Lancet. 2021;397(10290):2212-24. Epub 2021/04/25. doi: 10.1016/S0140-6736(20)32511-3. PubMed PMID: 33894145.

2. Corey KE, Kaplan LM. Obesity and liver disease: the epidemic of the twenty-first century. Clin Liver Dis. 2014;18(1):1-18. Epub 2013/11/28. doi: 10.1016/j.cld.2013.09.019. PubMed PMID: 24274861.

3. Raza S, Rajak S, Anjum B, Sinha RA. Molecular links between non-alcoholic fatty liver disease and hepatocellular carcinoma. Hepatoma Res. 2019;5:42. Epub 2019/12/24. doi: 10.20517/2394-5079.2019.014. PubMed PMID: 31867441; PubMed Central PMCID: PMCPMC6924993.

4. Doycheva I, Issa D, Watt KD, Lopez R, Rifai G, Alkhouri N. Nonalcoholic Steatohepatitis is the Most Rapidly Increasing Indication for Liver Transplantation in Young Adults in the United States. J Clin Gastroenterol. 2018;52(4):339-46. Epub 2017/09/30. doi: 10.1097/MCG.0000000000000925. PubMed PMID: 28961576.

5. Friedman SL, Neuschwander-Tetri BA, Rinella M, Sanyal AJ. Mechanisms of NAFLD development and therapeutic strategies. Nat Med. 2018;24(7):908-22. Epub 2018/07/04. doi: 10.1038/s41591-018-0104-9. PubMed PMID: 29967350; PubMed Central PMCID: PMCPMC6553468.

6. Rinella ME. Nonalcoholic fatty liver disease: a systematic review. JAMA. 2015;313(22):2263-73. Epub 2015/06/10. doi: 10.1001/jama.2015.5370. PubMed PMID: 26057287.

7. Younossi ZM, Koenig AB, Abdelatif D, Fazel Y, Henry L, Wymer M. Global epidemiology of nonalcoholic fatty liver disease-Meta-analytic assessment of prevalence, incidence, and outcomes. Hepatology. 2016;64(1):73-84. Epub 2015/12/29. doi: 10.1002/hep.28431. PubMed PMID: 26707365.

8. McGinley AM, Edwards SC, Raverdeau M, Mills KHG. Th17cells, gammadelta T cells and their interplay in EAE and multiple sclerosis. J Autoimmun. 2018. Epub 2018/02/06. doi: 10.1016/j.jaut.2018.01.001. PubMed PMID: 29395738.

9. Kullberg MC, Jankovic D, Feng CG, Hue S, Gorelick PL, McKenzie BS, et al. IL-23 plays a key role in Helicobacter hepaticus-induced T cell-dependent colitis. J Exp Med. 2006;203(11):2485-94. Epub 2006/10/13. doi: 10.1084/jem.20061082. PubMed PMID: 17030948; PubMed Central PMCID: PMCPMC2118119.

10. Yen D, Cheung J, Scheerens H, Poulet F, McClanahan T, McKenzie B, et al. IL-23 is essential for T cell-mediated colitis and promotes inflammation via IL-17 and IL-6. J Clin Invest. 2006;116(5):1310-6. Epub 2006/05/04. doi: 10.1172/JCI21404. PubMed PMID: 16670770; PubMed Central PMCID: PMCPMC1451201.

11. van der Fits L, Mourits S, Voerman JS, Kant M, Boon L, Laman JD, et al. Imiquimod-induced psoriasis-like skin inflammation in mice is mediated via the IL-23/IL-17 axis. J Immunol. 2009;182(9):5836-45. Epub 2009/04/22. doi: 10.4049/jimmunol.0802999. PubMed PMID: 19380832.

12. Parham C, Chirica M, Timans J, Vaisberg E, Travis M, Cheung J, et al. A receptor for the heterodimeric cytokine IL-23 is composed of IL-12Rbeta1 and a novel cytokine receptor subunit, IL-23R. J Immunol. 2002;168(11):5699-708. Epub 2002/05/23. doi: 10.4049/jimmunol.168.11.5699. PubMed PMID: 12023369.

13. Langrish CL, Chen Y, Blumenschein WM, Mattson J, Basham B, Sedgwick JD, et al. IL-23 drives a pathogenic T cell population that induces autoimmune inflammation. J Exp Med. 2005;201(2):233-40. Epub 2005/01/20. doi: 10.1084/jem.20041257. PubMed PMID: 15657292; PubMed Central PMCID: PMCPMC2212798.

14. Cua DJ, Sherlock J, Chen Y, Murphy CA, Joyce B, Seymour B, et al. Interleukin-23 rather than interleukin-12 is the critical cytokine for autoimmune inflammation of the brain. Nature. 2003;421(6924):744-8. Epub 2003/03/01. doi: 10.1038/nature01355. PubMed PMID: 12610626.

15. Murphy CA, Langrish CL, Chen Y, Blumenschein W, McClanahan T, Kastelein RA, et al. Divergent pro- and antiinflammatory roles for IL-23 and IL-12 in joint autoimmune inflammation. J Exp Med. 2003;198(12):1951-7. Epub 2003/12/10. doi: 10.1084/jem.20030896. PubMed PMID: 14662908; PubMed Central PMCID: PMCPMC2194162.

16. Romagnani S, Maggi E, Liotta F, Cosmi L, Annunziato F. Properties and origin of human Th17 cells. Mol Immunol. 2009;47(1):3-7. Epub 2009/02/06. doi: 10.1016/j.molimm.2008.12.019. PubMed PMID: 19193443.

17. Ivanov, II, McKenzie BS, Zhou L, Tadokoro CE, Lepelley A, Lafaille JJ, et al. The orphan nuclear receptor RORgammat directs the differentiation program of proinflammatory IL-17+ T helper cells. Cell. 2006;126(6):1121-33. Epub 2006/09/23. doi: 10.1016/j.cell.2006.07.035. PubMed PMID: 16990136.

18. Meng F, Wang K, Aoyama T, Grivennikov SI, Paik Y, Scholten D, et al. Interleukin-17 signaling in inflammatory, Kupffer cells, and hepatic stellate cells exacerbates liver fibrosis in mice. Gastroenterology. 2012;143(3):765-76 e3. Epub 2012/06/13. doi: 10.1053/j.gastro.2012.05.049. PubMed PMID: 22687286; PubMed Central PMCID: PMCPMC3635475.

19. Giles DA, Moreno-Fernandez ME, Divanovic S. IL-17 Axis Driven Inflammation in Non-Alcoholic Fatty Liver Disease Progression. Curr Drug Targets. 2015;16(12):1315-23. Epub 2015/06/02. doi: 10.2174/1389450116666150531153627. PubMed PMID: 26028039; PubMed Central PMCID: PMCPMC4929857.

20. Tang Y, Bian Z, Zhao L, Liu Y, Liang S, Wang Q, et al. Interleukin-17 exacerbates hepatic steatosis and inflammation in non-alcoholic fatty liver disease. Clin Exp Immunol. 2011;166(2):281-90. Epub 2011/10/12. doi: 10.1111/j.1365-2249.2011.04471.x. PubMed PMID: 21985374; PubMed Central PMCID: PMCPMC3219903.

21. Giles DA, Moreno-Fernandez ME, Stankiewicz TE, Cappelletti M, Huppert SS, Iwakura Y, et al. Regulation of Inflammation by IL-17A and IL-17F Modulates Non-Alcoholic Fatty Liver Disease Pathogenesis. PLoS One. 2016;11(2):e0149783. Epub 2016/02/20. doi: 10.1371/journal.pone.0149783. PubMed PMID: 26895034; PubMed Central PMCID: PMCPMC4760740.

22. Harley IT, Stankiewicz TE, Giles DA, Softic S, Flick LM, Cappelletti M, et al. IL-17 signaling accelerates the progression of nonalcoholic fatty liver disease in mice. Hepatology. 2014;59(5):1830-9. Epub 2013/10/12. doi: 10.1002/hep.26746. PubMed PMID: 24115079; PubMed Central PMCID: PMCPMC3975735.

23. Yan S, Wang L, Liu N, Wang Y, Chu Y. Critical role of interleukin-17/interleukin-17 receptor axis in mediating Con A-induced hepatitis. Immunol Cell Biol. 2012;90(4):421-8. Epub 2011/06/22. doi: 10.1038/icb.2011.59. PubMed PMID: 21691280.

24. Boland ML, Oro D, Tolbol KS, Thrane ST, Nielsen JC, Cohen TS, et al. Towards a standard diet-induced and biopsy-confirmed mouse model of non-alcoholic steatohepatitis: Impact of dietary fat source. World J Gastroenterol. 2019;25(33):4904-20. Epub 2019/09/24. doi: 10.3748/wjg.v25.i33.4904. PubMed PMID: 31543682; PubMed Central PMCID: PMCPMC6737317.

25. Drescher HK, Weiskirchen R, Fulop A, Hopf C, de San Roman EG, Huesgen PF, et al. The Influence of Different Fat Sources on Steatohepatitis and Fibrosis Development in the Western Diet Mouse Model of Non-alcoholic Steatohepatitis (NASH). Front Physiol. 2019;10:770. Epub 2019/07/12. doi: 10.3389/fphys.2019.00770. PubMed PMID: 31293441; PubMed Central PMCID: PMCPMC6603084.

26. Shih VF, Cox J, Kljavin NM, Dengler HS, Reichelt M, Kumar P, et al. Homeostatic IL-23 receptor signaling limits Th17 response through IL-22-mediated containment of commensal microbiota. Proc Natl Acad Sci U S A. 2014;111(38):13942-7. Epub 2014/09/10. doi: 10.1073/pnas.1323852111. PubMed PMID: 25201978; PubMed Central PMCID: PMCPMC4183330.

27. Matsumoto M, Hada N, Sakamaki Y, Uno A, Shiga T, Tanaka C, et al. An improved mouse model that rapidly develops fibrosis in non-alcoholic steatohepatitis. Int J Exp Pathol. 2013;94(2):93-103. Epub 2013/01/12. doi: 10.1111/iep.12008. PubMed PMID: 23305254; PubMed Central PMCID: PMCPMC3607137.

28. Younossi ZM, Blissett D, Blissett R, Henry L, Stepanova M, Younossi Y, et al. The economic and clinical burden of nonalcoholic fatty liver disease in the United States and Europe. Hepatology. 2016;64(5):1577-86. Epub 2016/10/22. doi: 10.1002/hep.28785. PubMed PMID: 27543837.

29. Albhaisi S, Noureddin M. Current and Potential Therapies Targeting Inflammation in NASH. Front Endocrinol (Lausanne). 2021;12:767314. Epub 2021/12/21. doi: 10.3389/fendo.2021.767314. PubMed PMID: 34925237; PubMed Central PMCID: PMCPMC8678040.

30. Gomes AL, Teijeiro A, Buren S, Tummala KS, Yilmaz M, Waisman A, et al. Metabolic Inflammation-Associated IL-17A Causes Non-alcoholic Steatohepatitis and Hepatocellular Carcinoma. Cancer Cell. 2016;30(1):161-75. Epub 2016/07/15. doi: 10.1016/j.ccell.2016.05.020. PubMed PMID: 27411590.

31. Schaalan MF, Mohamed WA, Amin HH. Vitamin D deficiency: correlation to interleukin-17, interleukin-23 and PIIINP in hepatitis C virus genotype 4. World J Gastroenterol. 2012;18(28):3738-44. Epub 2012/08/02. doi: 10.3748/wjg.v18.i28.3738. PubMed PMID: 22851868; PubMed Central PMCID: PMCPMC3406428.

32. Luci C, Bourinet M, Leclere PS, Anty R, Gual P. Chronic Inflammation in Non-Alcoholic Steatohepatitis: Molecular Mechanisms and Therapeutic Strategies. Front Endocrinol (Lausanne). 2020;11:597648. Epub 2021/01/02. doi: 10.3389/fendo.2020.597648. PubMed PMID: 33384662; PubMed Central PMCID: PMCPMC7771356.

33. Ghilardi N, Kljavin N, Chen Q, Lucas S, Gurney AL, De Sauvage FJ. Compromised humoral and delayed-type hypersensitivity responses in IL-23-deficient mice. J Immunol. 2004;172(5):2827-33. Epub 2004/02/24. doi: 10.4049/jimmunol.172.5.2827. PubMed PMID: 14978083.

Figure Legends

Figure 1. rmIL-23 administration induces RORγt proliferation in liver

FACS staining was performed of hepatic non-parenchymal cells (NPC) from mice administered either PBS or IL-23 by Intraperitoneal (IP) injections (A-G): Representative FACS gate, IL-23 induced greater frequency of RORγt$^+$Ki67$^+$ T Cells gated from CD3e$^+$CD4$^-$CD8$^-$ (A). Total RORγt$^+$Ki67$^+$ T Cells, indication of proliferative cell, and total RORγt (CD4-CD3-) T Cells quantified in the liver (B-C). Percentage quantification of Ki67+ of RORγt$^+$MAITs and RORγt$^+$ $\gamma\delta$ T Cells (D-E). Percentage of Neutrophils and Ly6c$^+$ monocytes from CD45+ Cells (F-G). Groups: Vehicle (PBS): n=6, IL-23 (3x 0.5ug): n=6. Data represents mean $\pm$ S.D. *p < 0.05, **p < 0.001, ****p < 0.0001, two-tailed t-test.

Figure 2. IL-23 is required for WD-induced hepatic IL-17 producing cell expansion

WT or Il23r-/- mice were fed a ND or WD for 20 weeks, followed by terminal analyses (A). FACS quantification of percent RORγt$^+$ from $\gamma\delta$ T Cells and RORγt$^+$ from MAIT cells (CD3e$^+$TCRb$^+$CD4$^-$CD8$^-$) (B-C). Percentage of IL-17A positive cells in $\gamma\delta$ T cells from WD fed WT and Il23r-/- liver non-parenchymal cells (NPCs) stimulated with Leukocyte Activation Cocktail with GolgiPlug (BD) for 4 hours (D). Groups: ND WT n=5, ND Il23r-/- n=5, WD WT n=7, WD Il23r-/- n=7. Data represents mean $\pm$ S.D. *p < 0.05, **p < 0.005, one-way ANOVA.

Figure 3. IL-23 signaling is dispensable for WD-induced hepatic inflammation

Liver sectioned H&E stained images of WT and Il23r-/- mice on ND or WD (A). Liver mRNA expression of *Tnf, Cd68, Ccl2, Cxcl2,* and *Cxcl10* (B). Liver FACS analysis of percent Ly6c$^+$

Monocytes and Neutrophils between the groups. Luminex results for serum levels of KC (E) and IP-10 (F) chemokines. Groups: ND WT n=5, ND Il23r-/- n=5, WD WT n=7, WD Il23r-/- n=7. Data represents mean ± S.D. *p < 0.05, **p < 0.005, ****p < 0.00005, one-way ANOVA. C/D??????

Figure 4. IL-23 signaling does not contribute to WD-induced hepatic fibrosis

Trichrome staining images of livers (A). Liver mRNA expression of *Col1a1, Col1a2,* and *Col3a1*. Groups: ND WT n=5, ND Il23r-/- n=5, WD WT n=7, WD Il23r-/- n=7.

Figure 5. IL-23 signaling does not contribute to WD-induced liver dysfunction

Body weights in gram(g) (A) and percent liver weights from body weight (B). Quantification of serum liver enzymes for Alanine Aminotransferase (ALT) (C), Aspartate Aminotransferase (AST) (D), and Alkaline Phosphatase (AP) (E) measured. Serum Albumin protein (F), serum cholesterol (G) and serum triglycerides (H) quantified. Groups: ND WT n=5, ND Il23r-/- n=5, WD WT n=7, WD Il23r-/- n=7. Data represents mean ± S.D, one-way ANOVA.

Figure 6. IL-23 signaling does not contribute to liver inflammation and fibrosis in the CDA-HFD NASH model.

WT and Il23r-/- mice were fed CDA-HFD for 9 weeks, followed by liver analysis (A). Percent quantification of hepatic RORγt in γδ T Cells (B) and MAITs (C). Trichrome staining images and quantification of Inflammation (E) and Trichrome Collagen content (F). Liver mRNA expression of *Tnf, Cd68, Ccl2, Cxcl2, Col1a1, Col1a2,* and *Col3a1* (G). Groups: ND WT n=3, ND Il23r-/- n=3, CDA-HFD WT n=6, CDA-HFD Il23r-/- n=6. Data represents mean ± S.D. ***p < 0.0005, one-way ANOVA.

Figure 7. IL-23 signaling does not contribute to CDA-HFD-induced liver dysfunction.

Body weights in gram (g) (A) and percent liver weights from body weight (B). Quantification of serum liver enzymes for Alanine Aminotransferase (ALT) (C), Aspartate Aminotransferase (AST) (D), and Alkaline Phosphatase (AP) (E) measured. Serum Albumin protein (F), serum cholesterol (G) and serum triglycerides (H) quantified. Groups: ND WT n=3, ND Il23r-/- n=3, CDA-HFD WT n=6, CDA-HFD Il23r-/- n=6. Data represents mean $\pm$ S.D.

Figure 1

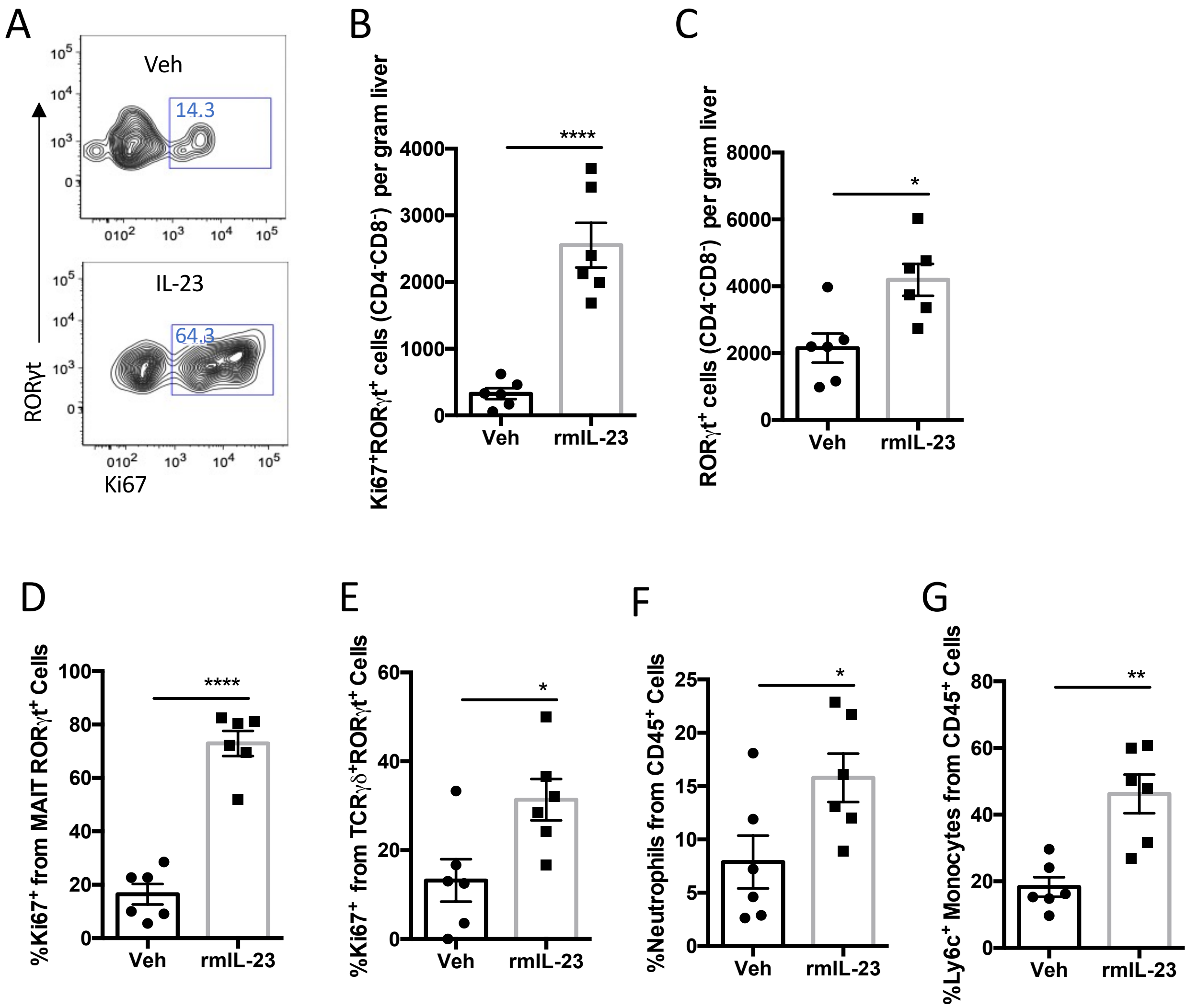
A
B
C
Veh
IL-23
14.3
64.3
RORγt
Ki67
Ki67⁺RORγt⁺ cells (CD4⁻CD8⁻) per gram liver
RORγt⁺ cells (CD4⁻CD8⁻) per gram liver
Veh
rmIL-23
****
*
D
E
F
G
%Ki67⁺ from MAIT RORγt⁺ Cells
%Ki67⁺ from TCRγδ⁺RORγt⁺ Cells
%Neutrophils from CD45⁺ Cells
%Ly6c⁺ Monocytes from CD45⁺ Cells
**

Figure 2

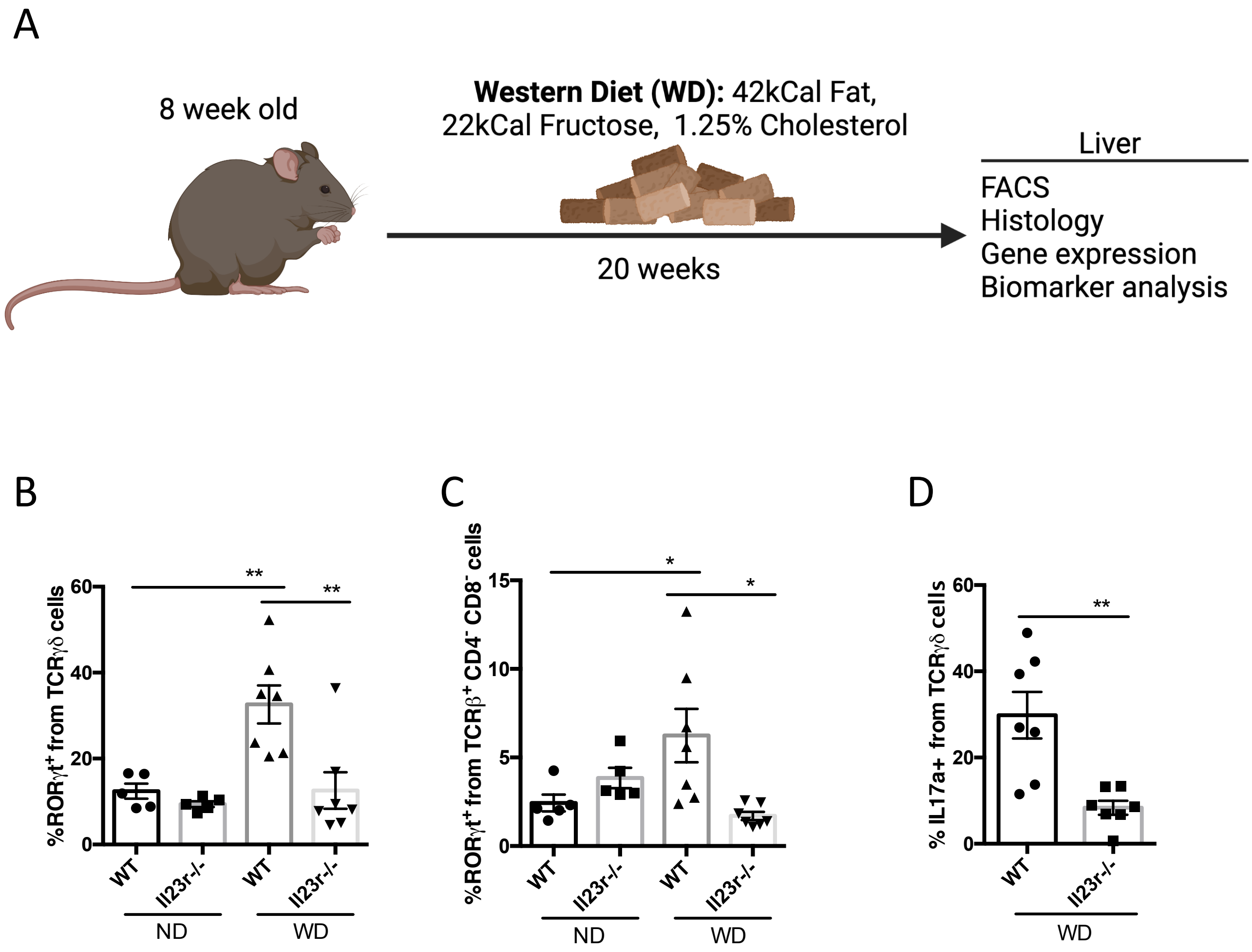

A
8 week old
Western Diet (WD): 42kCal Fat, 22kCal Fructose, 1.25% Cholesterol
20 weeks
Liver
FACS
Histology
Gene expression
Biomarker analysis
B
%RORγt+ from TCRγδ cells
60
40
20
0
**
**
WT
Il23r-/-
WT
Il23r-/-
ND
WD
C
%RORγt+ from TCRβ+ CD4- CD8- cells
15
10
5
0
*
*
WT
Il23r-/-
WT
Il23r-/-
ND
WD
D
% IL17a+ from TCRγδ cells
60
40
20
0
**
WT
Il23r-/-
WD

Figure 3

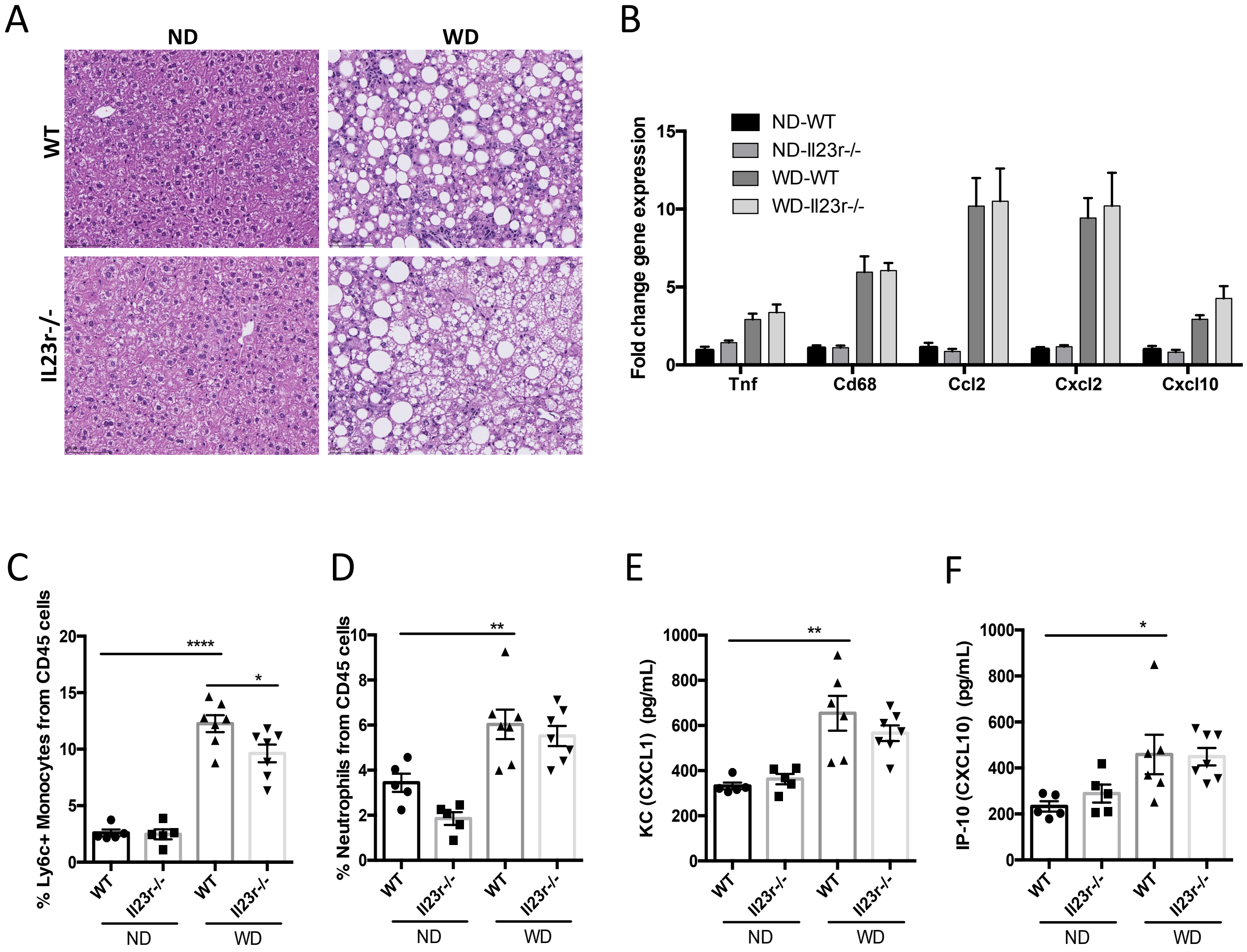

A
ND
WD
WT
IL23r-/-
B
ND-WT
ND-Il23r-/-
WD-WT
WD-Il23r-/-
Fold change gene expression
0
5
10
15
Tnf
Cd68
Ccl2
Cxcl2
Cxcl10
C
% Ly6c+ Monocytes from CD45 cells
0
5
10
15
20
****
*
WT
Il23r-/-
WT
Il23r-/-
ND
WD
D
% Neutrophils from CD45 cells
0
2
4
6
8
10
**
WT
Il23r-/-
WT
Il23r-/-
ND
WD
E
KC (CXCL1) (pg/mL)
0
200
400
600
800
1000
**
WT
Il23r-/-
WT
Il23r-/-
ND
WD
F
IP-10 (CXCL10) (pg/mL)
0
200
400
600
800
1000
*
WT
Il23r-/-
WT
Il23r-/-
ND
WD

Figure 4

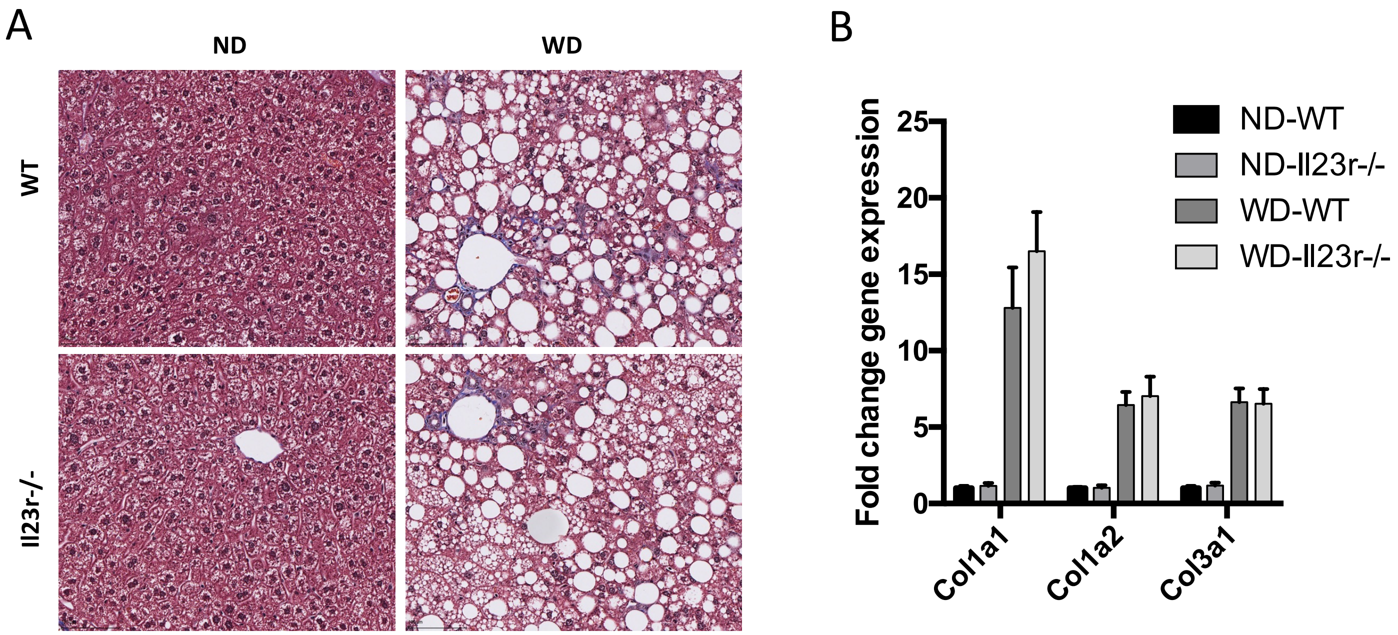
A
ND
WD
WT
Il23r-/-
B
Fold change gene expression
25
20
15
10
5
0
Col1a1
Col1a2
Col3a1
ND-WT
ND-Il23r-/-
WD-WT
WD-Il23r-/-

Figure 5

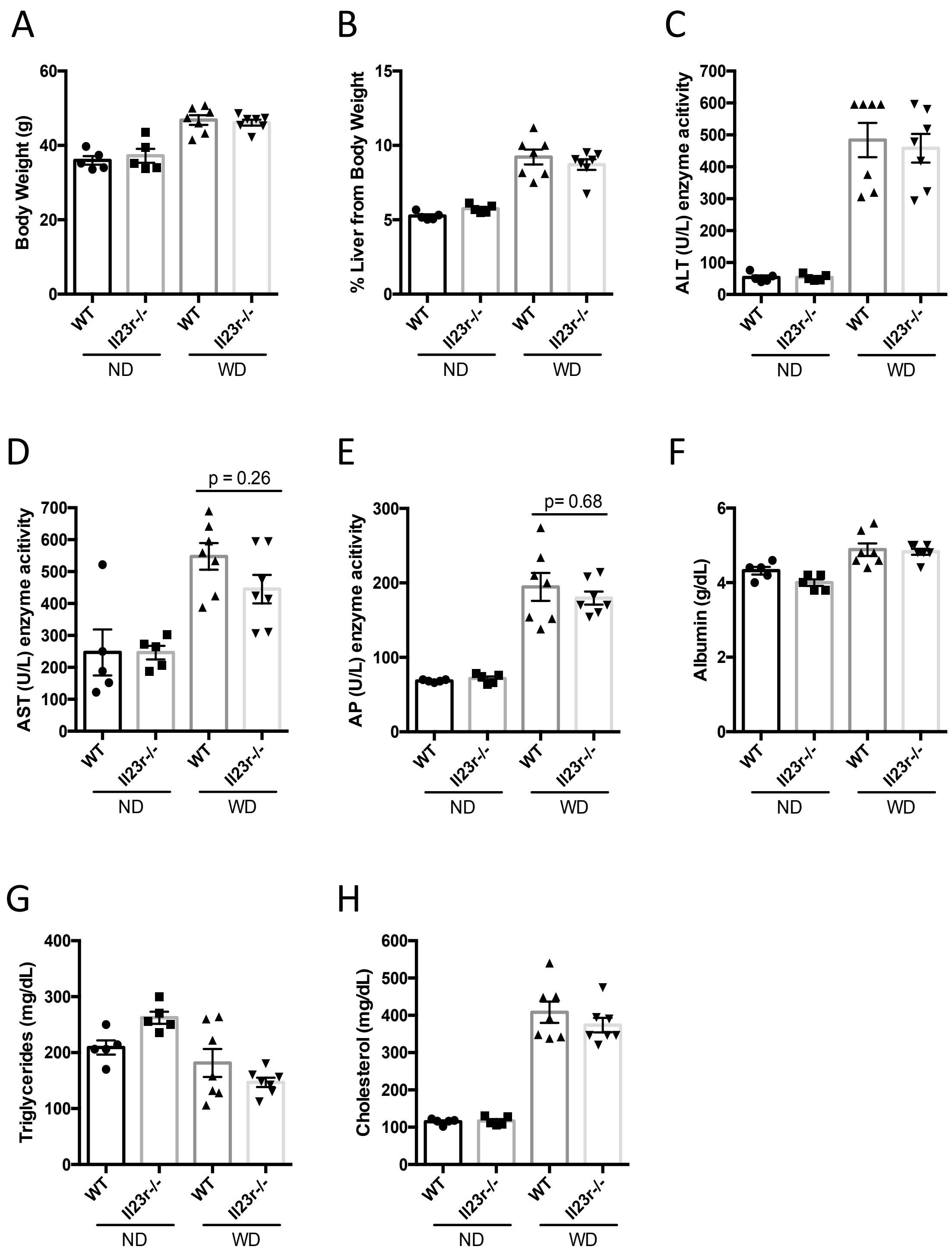

A
Body Weight (g)
B
% Liver from Body Weight
C
ALT (U/L) enzyme acitivity
D
p = 0.26
AST (U/L) enzyme acitivity
E
p= 0.68
AP (U/L) enzyme acitivity
F
Albumin (g/dL)
G
Triglycerides (mg/dL)
H
Cholesterol (mg/dL)
WT
Il23r-/-
ND
WD

Figure 6

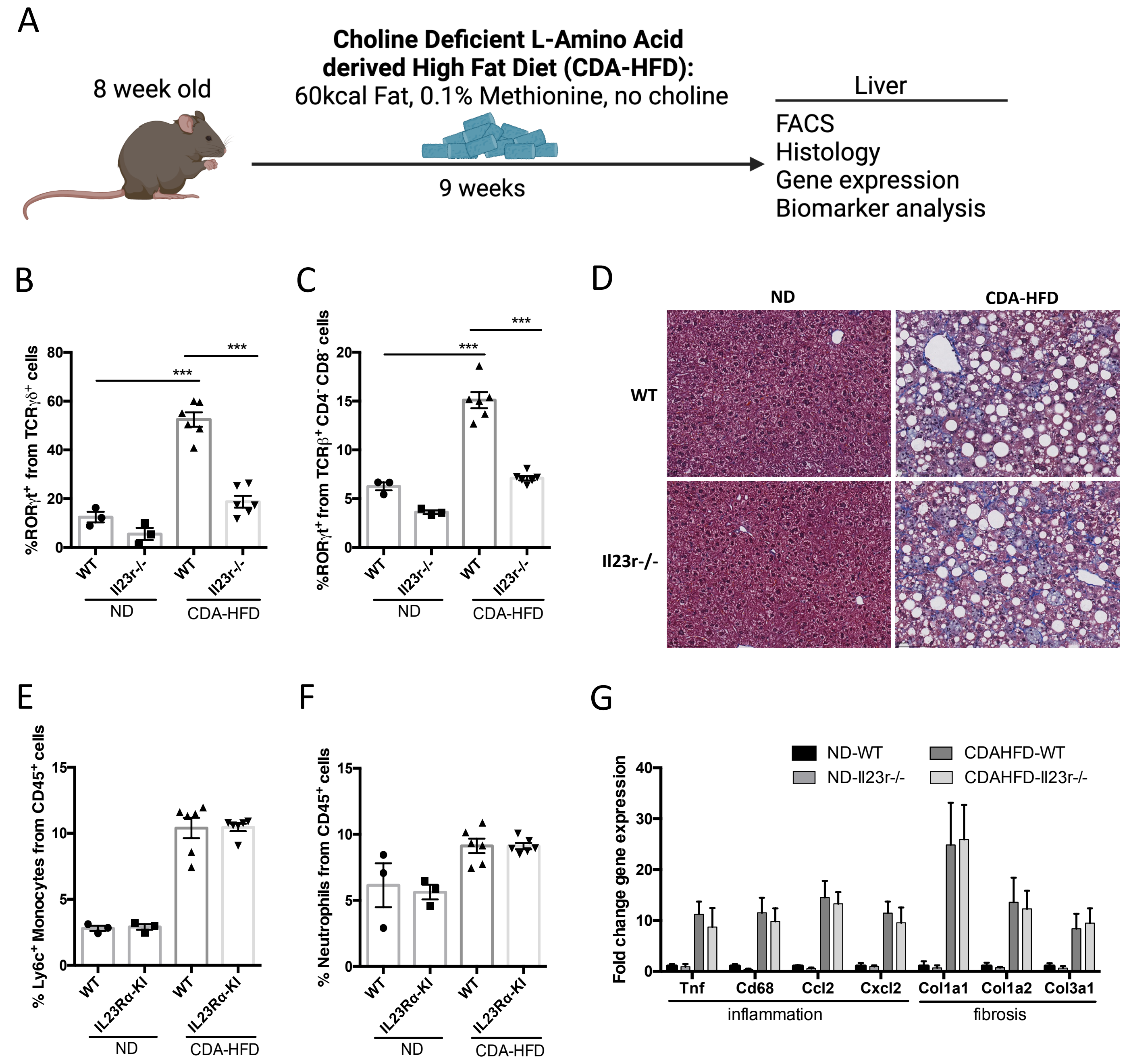
A
8 week old
Choline Deficient L-Amino Acid
derived High Fat Diet (CDA-HFD):
60kcal Fat, 0.1% Methionine, no choline
9 weeks
Liver
FACS
Histology
Gene expression
Biomarker analysis
B
C
D
ND
CDA-HFD
WT
Il23r-/-
E
F
G
ND-WT
ND-Il23r-/-
CDAHFD-WT
CDAHFD-Il23r-/-
Fold change gene expression
Tnf
Cd68
Ccl2
Cxcl2
Col1a1
Col1a2
Col3a1
inflammation
fibrosis
% Ly6c+ Monocytes from CD45+ cells
% Neutrophils from CD45+ cells
IL23Rα-KI
***

Figure 7

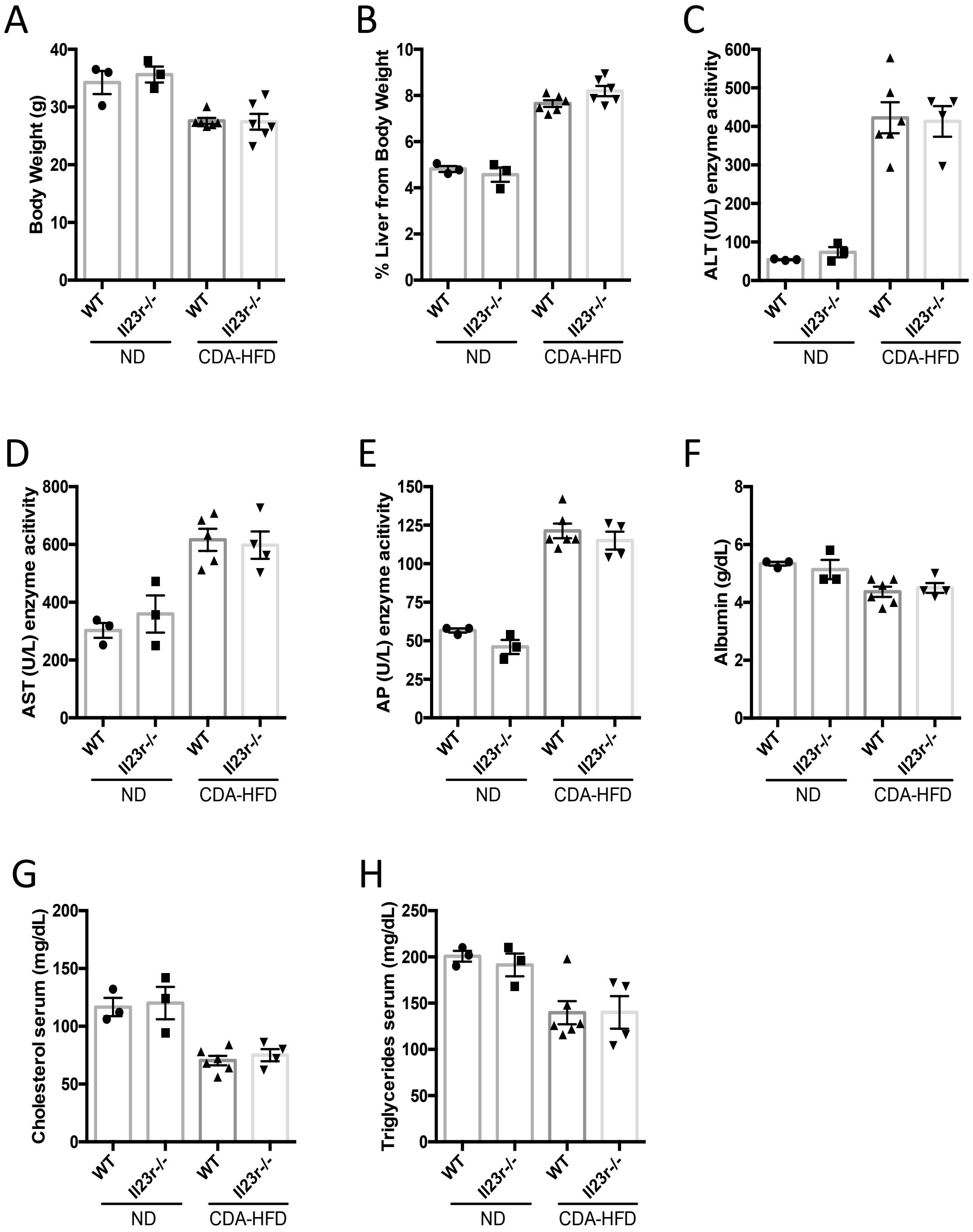
A
Body Weight (g)
B
% Liver from Body Weight
C
ALT (U/L) enzyme acitivity
D
AST (U/L) enzyme acitivity
E
AP (U/L) enzyme acitivity
F
Albumin (g/dL)
G
Cholesterol serum (mg/dL)
H
Triglycerides serum (mg/dL)
WT
Il23r-/-
ND
CDA-HFD
